# Classification of microarrays; synergistic effects between normalization, gene selection and machine learning

**DOI:** 10.1186/1471-2105-12-390

**Published:** 2011-10-07

**Authors:** Jenny Önskog, Eva Freyhult, Mattias Landfors, Patrik Rydén, Torgeir R Hvidsten

**Affiliations:** 1 Umeå Plant Science Center, Department of Plant Physiology, Umeå University, 901 87 Umeå, Sweden; 2 Department of Clinical Microbiology, Division of Clinical Bacteriology, Umeå University, 901 85 Umeå, Sweden; 3 Department of Mathematics and Mathematical Statistics, Umeå University, 901 87 Umeå, Sweden; 4 Computational Life Science Cluster (CLiC), Umeå University, 901 87 Umeå, Sweden; 5 Department of Medical Sciences, Uppsala University, Academic Hospital, 751 85 Uppsala, Sweden

## Abstract

**Background:**

Machine learning is a powerful approach for describing and predicting classes in microarray data. Although several comparative studies have investigated the relative performance of various machine learning methods, these often do not account for the fact that performance (e.g. error rate) is a result of a series of analysis steps of which the most important are data normalization, gene selection and machine learning.

**Results:**

In this study, we used seven previously published cancer-related microarray data sets to compare the effects on classification performance of five normalization methods, three gene selection methods with 21 different numbers of selected genes and eight machine learning methods. Performance in term of error rate was rigorously estimated by repeatedly employing a double cross validation approach. Since performance varies greatly between data sets, we devised an analysis method that first compares methods within individual data sets and then visualizes the comparisons across data sets. We discovered both well performing individual methods and synergies between different methods.

**Conclusion:**

Support Vector Machines with a radial basis kernel, linear kernel or polynomial kernel of degree 2 all performed consistently well across data sets. We show that there is a synergistic relationship between these methods and gene selection based on the T-test and the selection of a relatively high number of genes. Also, we find that these methods benefit significantly from using normalized data, although it is hard to draw general conclusions about the relative performance of different normalization procedures.

## Background

Machine learning methods have found many applications in gene expression data analysis, and are commonly used to classify patient samples into classes, corresponding to for example cancer sub-type, based on gene expression profiles. Supervised learning is a powerful tool in these studies since it can be used both to establish whether the classes of interest can be predicted from expression profiles and to provide an explanation as to what genes underlie the differences between classes. The expression data in such studies typically undergo an analysis pipeline in which the most important steps are data normalization, gene selection and machine learning. Although there are several comparative studies of methods for normalization, gene selection and machine learning, none have studied how all of these analysis steps influence each other and the final model performance.

A wealth of methods exists for microarray normalization, gene selection and machine learning. Normalization of microarray data involves several possible steps [1], including background correction [2] and dye-normalization [3]. The relative performance of different normalization approaches, although not in the context of machine learning, has previously been evaluated using spike-in data sets [4,5]. Previous studies have also shown that normalization has an impact on clustering [6,7]. One of the challenges in using machine learning and gene expression data to study medical diagnosis is the large number of genes (features) compared to the relatively limited number of patients (observations). Many gene selection methods have therefore been developed to cope with this problem [8,9]. Approaches to gene selections are either filter methods or wrapper methods. Filter methods score, rank and select the best individual genes before the machine learning methods is applied, while wrapper methods score subsets of genes according to the performance of machine learning models induced from the subset. Machine learning methods are commonly used in bioinformatics applications both for clustering (i.e. unsupervised learning) and for inducing predictive models from examples (i.e. supervised learning) [10].

Since gene selection is a necessary step in machine learning-based analysis of microarray patient data, all existing comparative studies have investigated the effect of gene selection and machine learning methods on classification performance. Most of these studies considered tumor classification. However, to the best of our knowledge, no study has also taken data normalization methods into account. Pirooznia *et al*. [11] studied the performance of three gene selection methods and six machine learning methods on eight microarray data sets, and mainly highlighted the importance of gene selection and the number of selected genes. Romualdi *et al*. [12] investigated four gene selection methods and six machine learning method using both simulated data and two microarray data sets, and demonstrated that non-parametric methods such as Support Vector Machines (SVMs) and Artificial Neural Networks (NNs) were more robust than parametric methods. Lee *et al*. [13] performed an extensive comparison of 21 machine learning methods and three gene selection approaches on seven microarray data sets. Their main conclusions were that more sophisticated classifiers such as SVMs perform better than classical methods and that the choice of gene selection method has a large effect on the performance. Li *et al*. [14] investigated eight gene selection methods and seven machine learning methods using nine data sets, and concluded that SVM methods perform best and that the choice of machine learning methods is more important than the choice of gene selection methods. Statnikov *et al*. [15] applied four gene selection methods and four machine learning methods, as well as several different SVM methods and some ensemble methods, to classify patients in 11 data sets. They also concluded that SVM methods performed better than non-SVM methods, that no significant improvement was obtained using ensemble methods and that gene selection improved all machine learning methods. All comparative studies used cross validation to evaluate the performance of different methods. Several previous studies have stressed the challenges related to the applications of machine learning methods and the importance of objective evaluation [16-18]. In particular, Zervakis *et al*. [19] used several gene selection and machine learning methods to show how the performance of gene selection methods vary with different validation strategies and concluded that independent test sets are important for validation.

The previous comparative studies have shown that classification performance varies a great deal from data set to data set. This is a challenge in comparative studies since it limits our ability to find general trends in terms of methods and combinations of methods that perform best across data sets. Here we approach this problem by comparing methods and pairs of methods on individual data sets, and then by visualizing trends across data set using heat maps. Thus we are able to draw general conclusions both about the individual effect of normalization, gene selection and machine learning on classification performance, and also to say something about synergistic effects that occur when these methods are used in combination. Our approach to studying synergy between methods, and the fact that we study the effect of normalization as well as gene selection and machine learning, makes our study unique. Our main conclusions are that Support Vector Machines (SVMs) with a radial basis kernel, linear kernel or polynomial kernel of degree 2 perform best across data sets. We show that these methods exhibit a synergistic relationship with gene selection based on the T-test and the selection of a relatively high number of genes. All these methods perform better on normalized than on non-normalized data, however, while the radial basis kernel and the linear kernel perform best when the data is not background corrected, the polynomial kernel benefit from background corrected data.

## Results

We evaluated classification models induced from seven different two-channel microarray expression data sets with two known classes (Table 1). Each classification model is the result of a combination of different computational methods for microarray normalization, gene selection, number of selected genes and machine learning. We included five different approaches to normalization, three gene selection methods, 21 different numbers of genes and eight different machine learning methods (Table 1). In total 14685 models were induced corresponding to all possible combinations of the different methods and data sets. We will refer to data sets and methods according to the acronyms given in Table 1 (see Methods for more details).

**Table 1:** Overview of the data sets and the methods used in this study

Data set (D)	Classes*	No. of genes^**^
Alizadeh	DLBCL (68), other samples (65)	7806 (7430)
Finak	Epithelial (34), stromal tissue (32)	33491
Galland	Invasive NFPAs (22), non- invasive NFPAs (18)	40475 (40291)
Herschkowitz	High ER expression (58), low ER expression (46)	19718
Jones	Cancerous samples (72), non-cancerous samples (19)	40233 (39746)
Sørlie	High ER expression (55), low ER expression (18)	8033 (7734)
Ye	Metastatic (65), non-metastatic (22)	8911

**Normalization (No)**	**Description**	

No 0	Raw data	
No 1	Print-tip MA-loess, no background correction	
No 2	Print-tip MA-loess, background correction	
No 3	Global MA-loess, no background correction	
No 4	Global MA-loess, background correction	

**Gene selection (G)**	**Fixed parameters**	

T-test	Two-sided	
Relief	Threshold = 0, nosample = # obs. in data set	
Paired distance	Euclidian distance	

**Number of genes (N)**	2, 12, 22, 32, 42, 52, 62, 72, 82, 92, 100, 200, 300, 400, 150, 500, 600, 700, 800, 900, 1000	

**Machine learning (M)**	**Description, Fixed parameters**	**Optimized parameters**

DT Gini	Decision tree, Splitting index = Gini	
DT Information	Decision tree, Splitting index = Information	
NN One layer	Neural Network, one hidden layer, decay = 0.001, rang = 0.1, maxit = 100	size = [2-5]
NN No layer	Neural Network, no hidden layer, decay = 0.001, rang = 0.1, maxit = 100, skip = TRUE, size = 0	
SVM Linear	Support Vector Machine, linear kernel, type = nu-scv, cross = 10, nu = 0.2, scaled = FALSE	
SVM Poly2	Support Vector Machine, polynomial kernel, deg 2, type = nu-scv, cross = 10, nu = 0.2, scaled = FALSE	
SVM Poly3	Support Vector Machine, polynomial kernel, deg 3, type = nu-scv, cross = 10, nu = 0.2, scaled = FALSE	
SVM Rb	Support Vector Machine, radial basis kernel, type = nu-scv, cross = 10, nu = 0.2, scaled = FALSE	sigma = [2^-14^, 2^14^]

Acronyms defined here are used throughout the paper. "Fixed parameters" in the methods were given fixed values, while "Optimized parameters" were optimized in the inner cross validation using a grid search. *The number of samples belonging to each class is given in parenthesis. **Dimensions after background corrected normalization (No 2 and No 4) are given in parenthesis.

**Figure 1:**
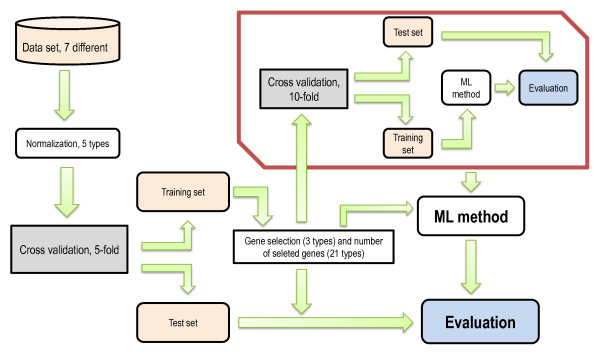
**Method overview**. The figure illustrates the analysis pipeline used to induce and validate models. First data was normalized (or raw data are used: No 0). Then a 5-fold cross validation (CV) was conducted to divide data into training and test sets. The training sets were used to train the models (red box), while the test sets were used to validate their classification power. In order to induce a classification model, some parameters had to be tuned. For example, the different Support vector machines (SVMs) employed different kernels with one or more parameters. The parameter sigma in the radial basis kernel was tuned by conducting a grid search and choosing the values with the lowest error rate during a 10-fold CV. The selected parameter value was finally used to induce a model from the training set in the outer CV and to classify the observations in the corresponding test set. The outer 5-fold CV was performed 10 times resulting in 50 test sets from which we evaluated 50 different models trained on 50 different trainings sets. As a measure of classification performance we used the average fraction of misclassified observations (i.e. error rate) in these 50 test sets.

Combinations of methods were validated by employing a double cross validation (CV) approach where the inner loop was used to find good parameter settings (e.g. number of units in the hidden layer of a Artificial Neural Network (NN)) and the outer loop was used to estimate the predictive power of the final model in terms of average error rate (Figure 1). Normalization was done initially, before the cross validation, while gene selection was done inside the outer cross validation.

### Method choices explain variation in classification performance within data sets

**Figure 2:**
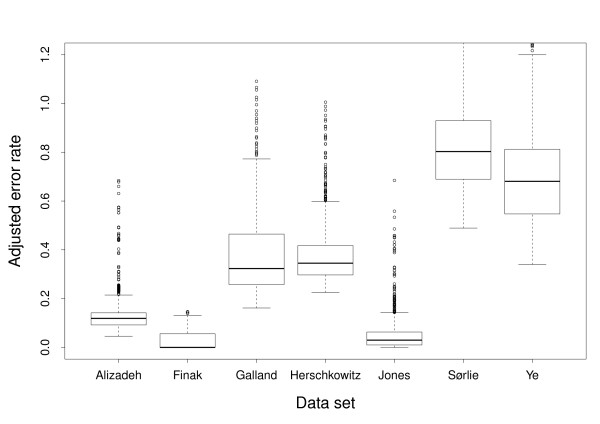
**Overall error rates for each data set**. Box plots showing the error rates resulting from all the different combinations of methods applied to the seven data sets.

The average error rate obtained by evaluating all the different combinations of methods on the seven data sets was 0.1512 with a standard deviation of 0.1195 (Additional file 1). The observed variation has two causes; the varying performance of different combination of methods used to induce models and the varying difficulty of discriminating the two classes in different data sets. The later could to some degree be explained by the varying distributions of observations over the two classes in the data sets. To reduce this effect, error rates were adjusted by dividing by the theoretical error rate obtained by random class assignment from the known distribution of classes. Thus adjusted error rates below one correspond to models performing better than random class assignment. Henceforth, when we discuss error rates, we will refer to the adjusted version of error rates if not specifically stated otherwise. The average adjusted error rate from all the 14685 models was 0.3668 with a standard deviation of 0.3159. This indicates that, although the adjustment reduces the influence due to unbalanced class sizes, most of the variation has other causes. Figure 2 shows that the error rates vary a lot between different data sets, but also that there is variation within data sets. It is this latter variation, due to the choice of combinations of methods, that is of interest to us in this paper.

To analyze the effect that different methods have on the classification performance, we used multiple linear regression [20] with methods as predicting variables and error rate as the response variable. Predicting variables included both first order terms and second order interaction terms constructed from Data set (D), Normalization (No), Gene selection method (G), Number of selected genes (N) and Machine learning method (M) (see Table 1). The regression model had an adjusted R-squared [20] value of 0.9589. The analysis showed that Data set (D) is by far the most explanatory predicting variable completely overshadowing the other variables. This is in accordance with what we already saw in Figure 2, and provides little information as to what is the best combination of methods. Thus, we performed a new regression analysis without Data set (D) as predicting variable, resulting in an adjusted R-squared value of only 0.0283. As a consequence of the lack of general, interpretable results from the regression analysis across data sets, we changed strategy to first analyze each data set individually and then search for general trends across data sets. This seems to be a viable strategy; adjusted R-squared values for these new regression models from the seven individual data sets range from 0.74 to 0.97, indicating that variation in error rate within data sets indeed can be explained by method choice. Also, all the first order terms (except G in one data set) and most interaction terms (except N+G and N+M in one data set) have significant explanatory power in the regression models (see Methods).

### Significant differences in performance of individual methods

**Figure 3:**
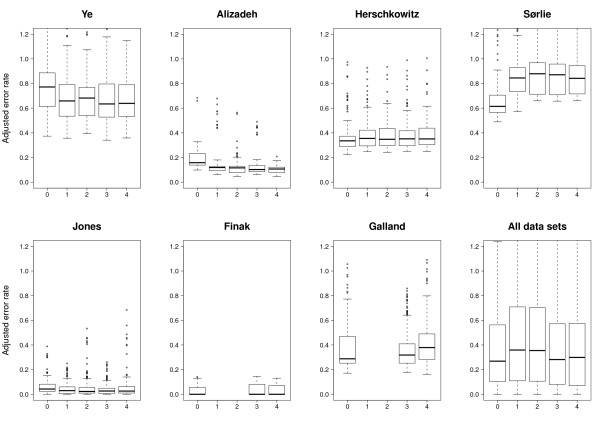
**Classification performance relative to normalization method**. Box plots showing the error rates resulting from all method-combinations utilizing a specific normalization method. Since the Finak and Galland data sets could not be normalized with methods 1 and 2, these methods are absent from the relevant plots.

**Figure 4:**
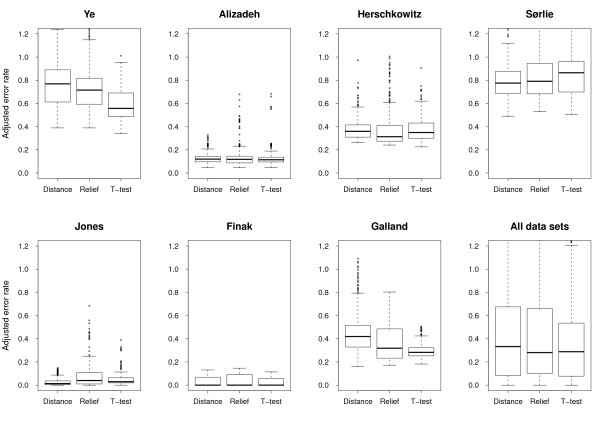
**Classification performance relative to gene selection method**. Box plots showing the error rates resulting from all method-combinations utilizing a specific gene selection method.

**Figure 5:**
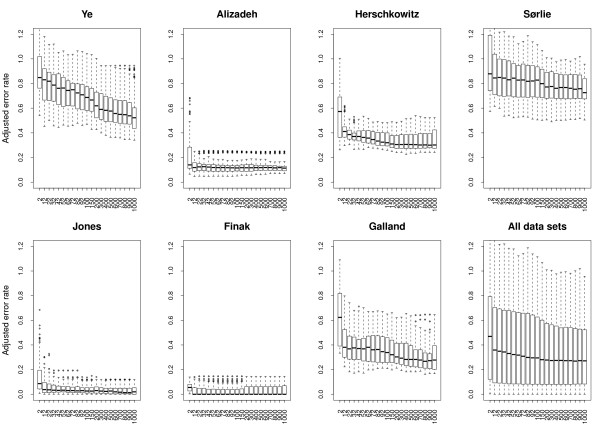
**Classification performance relative to different numbers of genes**. Box plots showing the error rates resulting from all method-combinations utilizing a specific number of genes.

**Figure 6:**
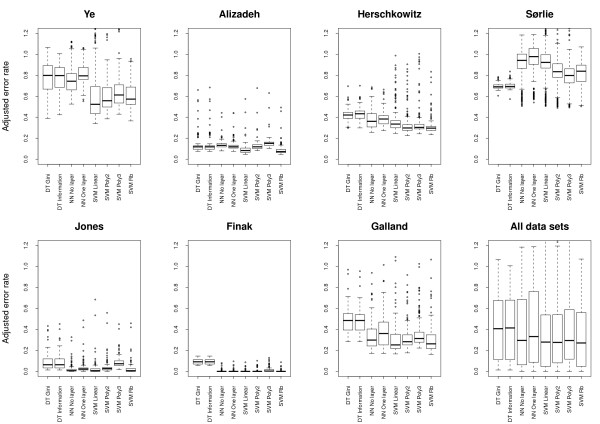
**Classification performance relative to machine learning method**. Box plots showing the error rates resulting from all method-combinations utilizing a specific machine learning method.

**Figure 7:**
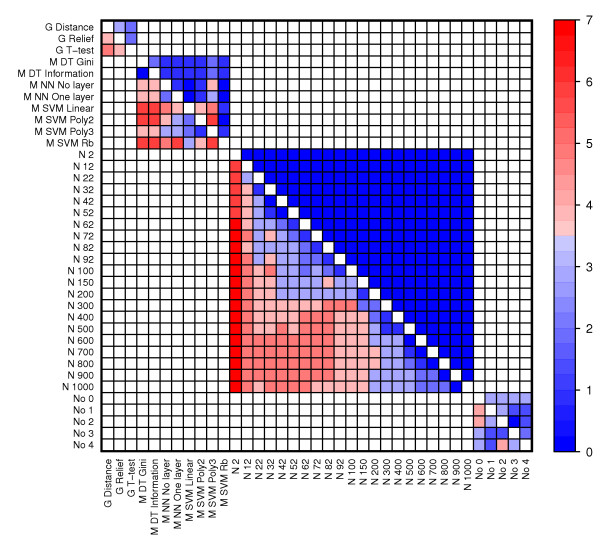
**The predictive performance of individual methods across data sets**. The heatmap visualize the number of data sets in which one method (row) performed significantly better than another method (column). The Wilcoxon signed-rank test was used to compare the error rates of all combinations containing one method against the error rates of all combinations containing the other method. Significance was determined using a Bonferroni corrected p-value threshold (i.e. 0.05 divided by the number of tests).

To study the performance of individual methods, e.g. the gene selection method Relief, we plotted error rates resulting from applying all relevant combinations of methods to individual data sets and to all data sets (Figure 3, Figure 4, Figure 5 and Figure 6). We also compared all methods head-to-head and visualized statistically significant differences between pairs of methods across data sets (see Figure 7, Additional file 2 and Methods).

#### Normalization

Across data sets, the normalization methods perform rather similar (Figure 3); the somewhat worse results for No 1 and 2 (i.e. print-tip normalization) can be explained by the fact that these normalization methods are not relevant to the Agilent data sets (i.e. Finak and Galland) of which the Finak data set results in extremely well-performing models for almost all methods (Figure 2). Within data sets, however, the picture is rather complex (Figure 3 and Figure 7). In the Sørlie data set, models based on non-normalized data (No 0) somewhat surprisingly outperform models from normalized data (No 1-4) by a large margin, but non-normalized data is also significantly better than all normalized data in Herschowitz. On the other hand, all normalized data is statistically better than non-normalized data in Ye, Alizadeh and Jones. Thus the major differences are observed between non-normalized and normalized data. Significant differences between normalization methods (No 1-4) are sporadic, although No 4 is significantly better than No 2 and No 3 in three data sets.

#### Gene selection

Also gene selection methods exhibit a rather complex pattern with some data sets showing a gene selection preference (Figure 4 and Figure 7). The T-test outperforms the two other methods in Ye and Galland, and is significantly better than Paired distance and Relief in five and four data sets, respectively. Relief is significantly better than Paired distance in Alizadeh, Galland, Herschkowitz and Ye. However, Paired distance is significantly better than the two others in Jones and Sørlie. Hence, while the general trend is that the T-test performs best followed by Relief and Paired distance, the Sørlie data set again shows the opposite trend.

#### Number of selected genes

Figure 5 shows that there is a clear improvement in error rates when going from selecting only the two best genes from the gene selection methods to selecting several genes. We also see that, for several data sets including Ye, Sørlie and Galland, more genes imply better performance, although this trend decreases as we add many more genes than we have observations. From Figure 7 it is clear that there is no statistically significant improvement in a majority of the data sets from including more than 200 genes. Since we are applying the cross validation approach, and thus estimate error rates on data that is unseen by the models, we expected the predictive power of the models to suffer somewhat from including many genes. What we instead observe is that the performance keeps improving or stays constant, but never gets worse. Interestingly, the three data sets that keep improving even when more than 200 genes are used (i.e. Ye, Sørlie and Galland) also have the fewest number of differentially expressed genes among all data sets (Table 2).

**Table 2:** Differently expressed genes in the datasets

Data set (D)	No. significant genes	Significance threshold
Alizadeh	787	6.40e-06
Finak	2145	1.49e-06
Galland	209	1.24e-06
Herschkowitz	324	2.54e-06
Jones	6282	1.24e-06
Sørlie	0	6.22e-06
Ye	47	5.61e-06

Number of significant genes in each data set and the corresponding significance thresholds. The t-test was used to compute p-values for each gene and the Bonferroni correction was used to judge significance (i.e. significance threshold at 0.05 divided by the number of genes).

#### Machine learning

In general, Support Vector Machines (SVMs) have the best classification capabilities followed by Artificial Neural Networks (NNs), while Decision Trees (DTs) have the worst performance (Figure 6 and Figure 7). However, DTs clearly outperform other machine learning methods in Sørlie, which again shows the opposite trend of the other data sets. Although there are some clear conclusions to be drawn from the performance of machine learning methods, Figure 7 also portray a rather complex picture where most methods perform significantly better than most other methods in at least one data set. Notably, SVMs with a radial basis (Rb) kernel are only outperformed in single data sets, but SVM Linear and SVM Poly 2 are also performing well.

### Synergistic relationships exists between methods

**Figure 8:**
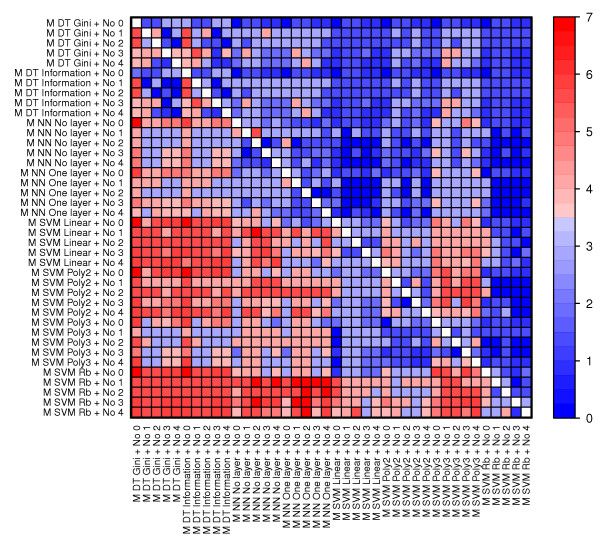
**The predictive performance of machine learning and normalization methods across data sets**. The heatmap visualize the number of data sets in which one pair of methods (row) performed significantly better than another pair of methods (column). The Wilcoxon signed-rank test was used to compare the error rates of all combinations containing one pair against the error rates of all combinations containing the other pair. Significance was determined using a p-value threshold of 0.05.

**Figure 9:**
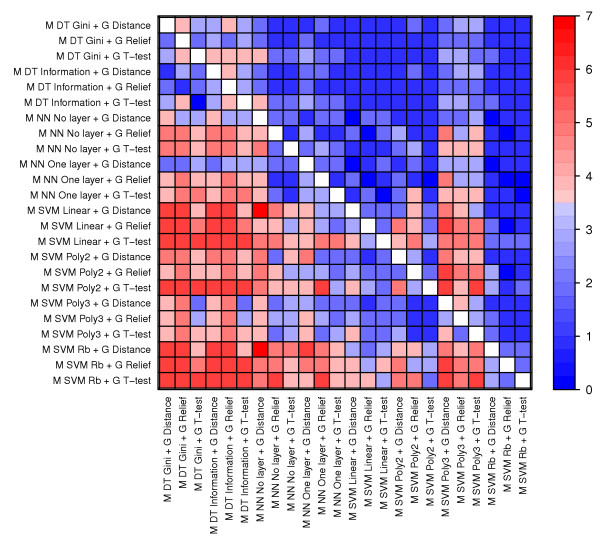
**The predictive performance of machine learning and gene selection methods across data sets**. See figure text of Figure 8.

**Figure 10:**
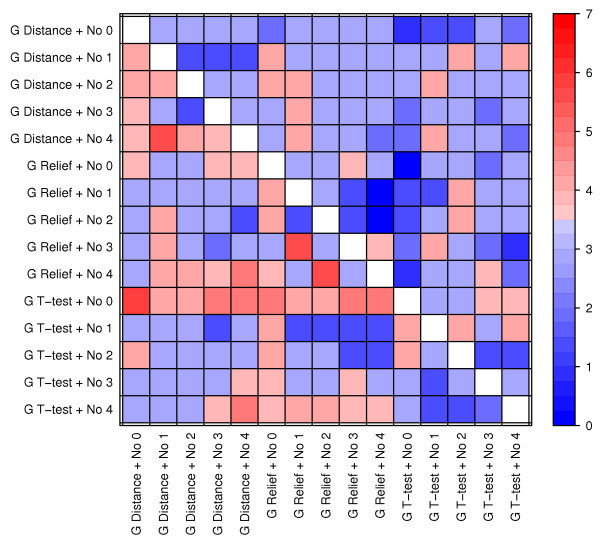
**The predictive performance of gene selection and normalization methods across data sets**. See figure text of Figure 8.

One of the main questions we ask in this study is to what degree we can observe synergistic relationships between methods. In order to answer this question we need to study combinations of methods. To have enough data to claim statistical significance, we limited our study of synergy to comparing all method-pairs of the same type, i.e., machine learning-normalization (M-No), machine learning-number of genes (M-N), machine learning-gene selection (M-G), gene selection-normalization (G-No), gene selection-number of genes (G-N) and normalization-number of genes (No-N) (see Figure 8, Figure 9, Figure 10, Additional file 3 and Methods).

Obviously, the performance of individual methods affects the performance of pairs of methods. However, in practice we always select one method of each type (i.e. one method for normalization, gene selection, number of genes and machine learning), thus methods that do not significantly deteriorate the performance of other individually well-performing methods are of interest as well as methods that decrease performance of other methods and therefore represent combinations that should be avoided. Method-pairs that contain the number of selected genes (N) are generally exhibiting the pattern already seen for single methods; too few genes severely reduce the performance of most methods while more than 200 genes do not significantly improve performance. Thus we will mainly focus on pairs consisting of methods for machine learning, normalization and gene selection (all comparisons can be seen in Additional file 3). Furthermore, we are particularly interested in pairs where the best machine learning methods (SVM Rb, Linear and Poly 2) are significantly affected by normalization or gene selection. The overall performance of a method-pair can be summarized by counting the number of data set where this pair is significantly better than other pairs (i.e. the sum of one row in Figure 8, Figure 9 and Figure 10, see Additional file 3 for these calculations).

#### Machine learning and normalization (Figure 8)

 Compared to all other pairs of machine learning and normalization, pairs containing SVM Rb perform the best. SVM Rb performs better on normalized than on non-normalized data. The best normalization method to use with SVM Rb is No 3 followed by No 1, although the improvement over No 2 and No 4 is rather small. The same pattern can be seen for the second best machine learning method, SVM Linear, indicating that these methods combine well with normalization methods not utilizing background correction (i.e. No 1 and No 3). However, the picture is rather complex. For example, the third best method, SVM Poly 2, works best with background corrected normalizations (No 2 and No 4), while normalizations without background correction is no better than non-normalized data.

#### Machine learning and gene selection (Figure 9)

 A clear trend is that the T-test is the best gene selection partner to all machine learning methods, although the actual improvement sometimes is rather small. SVM Rb combined with the T-test is the best performing method-pair, and T-test is also the best gene selection partner for SVM Linear and SVM Poly 2.

#### Gene selection and normalization (Figure 10)

 Almost all pairs of gene selection and normalization methods are significantly better than almost all other pairs of this type in at least two data sets. The lack of clear trends is maybe not so surprising considering the complex behavior of these methods when studied individually. Surprisingly, there is a synergy between the T-test and non-normalized data, which is the best pair of gene selection and normalization. If we look at the performance of the normalization methods for each gene selection method separately, we again see the trend from our analysis of individual methods; three to four data sets result in significantly better models when normalized while at least two other data sets actually result in better models when not normalized.

The comparison of method-pairs confirms the strong performance of the individually best performing machine learning methods (SVMs Rb, Linear and Poly2). These methods require at least 150 genes to achieve their best classification performance. A trend is that SVM Rb is somewhat more robust with respect to normalization, gene selection and number of selected genes than the other well-performing machine learning methods. For example, both SVM Linear and Poly 2 perform worse when not using gene selection based on the T-test, while SVM Rb performs almost as well with one of the two other methods. This example illustrates a very important trend in our study; although some methods perform well by themselves, their performance can be severely hampered by unfortunate choices for the other methods. In this context, data normalization is shown to be very important; the wrong normalization method can severely reduce the performance of many of the best machine learning methods. Also, these best methods perform better on normalized than non-normalized data. There is also a very clear positive effect of using normalized data with DT methods, while NN methods actually perform best on non-normalized data.

Of particular interest are synergistic effects where two methods perform significantly better together than any of the two methods do individually. We specifically searched for such patterns (Additional file 4), and confirmed synergistic relationships between the three best machine learning methods (SVM Rb, Linear and Poly2) and both the T-test (significant in at least three datasets) and the selection of at least 200 genes (significant in at least four data sets). There is also synergy between No 3 (normalization without background correction) and both SVM Rb and Linear (significant in three data sets), while SVM Poly2 has a synergistic relationship with background corrected data (No 2 and 4, significant in three and four data sets, respectively). One surprise is that this analysis confirms synergy between non-normalized data and the T-test (significant in four data sets). However, since the best machine learning methods perform well with normalized data and the T-test, this result is a curiosity of little practical importance.

#### Statistical significance

**Figure 11:**
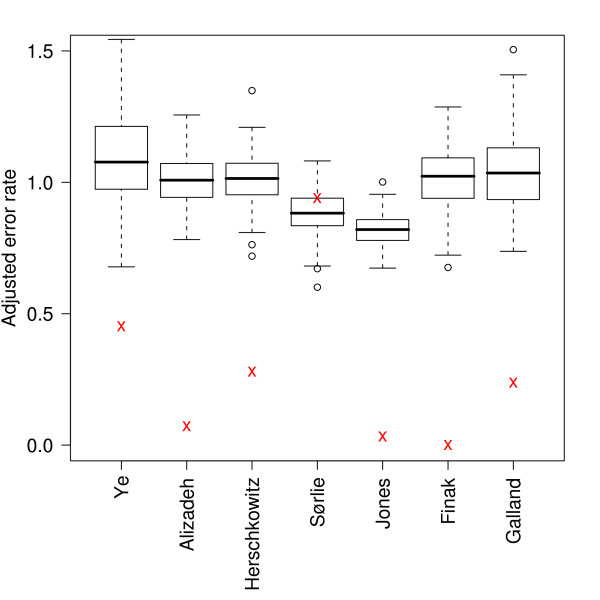
**Permutation tests**. The plots show the spread of adjusted error rates resulting from randomly shuffling class labels in each data set. The results were obtained using the method combination No 3, T-test, 150 genes and SVM Rb, and each data set was permutated 300 times. Adjusted error rates from the original class labels are marked by red crosses.

To establish statistical significance of the reported error rates, we performed permutation tests by inducing models and estimating error rates after randomly shuffling the class labels of all data sets (Figure 11). Since such tests are computationally expensive, we limited the study to one method combination that was chosen based on earlier discussions related to performance and synergy; No 3, T-test, 150 genes and SVM Rb. The permutation tests show that shuffled data sets result in adjusted error rates centered close to 1.0 and that our reported error rates for the original data sets are highly significant with the exception of Sørlie. As discussed earlier, the Sørlie dataset stands out from the other data sets in many ways. For example, Sørlie results in better models when non-normalized and when inducing models using decision trees.

## Discussion

In this paper, we have studied the effect of data normalization, gene selection and machine learning on the predictive performance of models induced from cancer-related expression data. Performance was rigorously assessed by repetitively employing a double cross validation approach to each method combination and each data set. We analyzed seven cancer related two-channel microarray data sets published in high-impact journals. We were particularly interested in studying the effect of normalization in two-channel experiments where a generally agreed-upon standard for normalization still does not exist. Indeed we see some trends related to normalization. Normalized data resulted in better models than do non-normalized data when employing the best machine learning methods. In particular, the two best machine learning methods (SVM Rb and Linear) showed a synergistic relationship with normalizations not using background correction (i.e. No 1 and No 3).

The data sets in this study result in machine learning models that perform rather differently both with respect to error rate and also with respect to relative performance between methods. Although all data sets are from two-channel microarray experiments related to cancer, the classes we have used to train and evaluate our models are defined somewhat differently. However, all classes are based on clinical observations (for details see Table 1). We chose to approach this challenge of heterogeneous data sets and classes by initially evaluating the data sets separately and then by looking for general trends across data sets.

Overfitting occurs when the learning framework selects models that performs better on the training set but worse on the external test sets [21]. This is a particularly severe problem for the types of data sets studied here, since we have many more genes than patients, and thus a high risk of selecting genes that discriminate classes in the training set but that do not generalize to the test set [17]. To detect overfitting and obtain robust results, we performed a double cross validation where the inner loop was used to optimize parameters (when applicable) and the outer loop was used to estimate classification performance. A k-fold CV was chosen since previous research has shown that this reduce the bias compared to leave-one-out CV [22]. In addition, we re-ran the CV several times to minimize the effect that particular data splits have on the results.

To simultaneously study the effect of normalization, gene selection and machine learning implies testing a large number of method combinations. In order to reduce running time, we therefore had to make certain adjustments to the analysis pipeline used to induce and validate models. Firstly, while we did gene selection inside the outer CV loop, we chose not to perform a separate selection inside the inner loop (see Figure 1). Since the inner loop is used to optimize parameters only, we found this to be a reasonable compromise to reduce running time while still keeping training and test sets completely separate when estimating the reported error rates in the outer CV loop. Secondly, we decided to fix several parameters in the machine learning methods. Although we carried out tests to make sure we optimized the most important parameters, this approach could give some advantage to methods that have parameters that were in fact optimized. For example, optimizing the Gaussian kernel parameter σ could somewhat benefit SVM Rb since this is the only SVM method left with a parameter that was tuned. Finally, we only performed permutation tests for one method combinations (300 permutations with No 3, T-test, 150 genes and SVM Rb). However, this test was sufficient to show that permutated data results in adjusted error rates centered close to 1.0 and thus that the best methods in this study clearly produce statistically significant results.

Due to the above mentioned risk of overfitting, and also due to practical issues such as running time, gene selection is required on data sets with many genes and few observations (e.g. patients). We were rather surprised to see that all data sets upheld predictive performance even when 1000 genes were selected from the gene selection methods, and that the error rate actually improved for three data sets. Interestingly, the three data sets for which performance continuously improved when more genes were selected, were the same three data sets that have the fewest number of discriminatory genes (Table 2); Ye, Sørlie and Galland. These data sets are also among the four worst performing data sets in terms of average error rate (Galland is slightly better than Herschkowitz). Thus, one might conclude that the inclusion of many genes give better performance for data sets with many weakly discriminatory genes as compared to data sets with strongly discriminatory genes. It was also encouraging to see that our training pipeline seems robust to overfitting in that the inclusion of weakly discriminatory genes (most data sets have fewer than 1000 significantly discriminatory genes, Table 2) did not affect classification performance negatively for any data set.

The gene selection methods considered in this study were all filter-based methods. These methods select genes prior to machine learning by typically ranking genes based on their individual ability to separate classes [9]. Unfortunately, this reduces the possibility for advanced non-linear machine learning methods to find complex discriminatory patterns or decision boundaries based on genes that individually are weakly discriminatory or even completely non-discriminatory. Wrapper-based gene selection methods can in principle find such genes by iteratively testing subsets of genes that result in high-performing models [9]. However, for the data sets that we are studying here, with tens of thousands of genes and only around 100 patients, wrapper methods are unrealistic not only in terms of time complexity, but also due to the risk of overfitting. In this context, we designed a new gene selection method (called Paired distance) that first selects genes with high variance or high absolute mean, and then ranks pairs of genes with high discriminatory power (see Methods). Our hope was that this method would identify discriminatory gene-pairs containing genes that individually were not selected by other gene selection methods and thus would improve models from data sets with only weakly discriminatory genes. Intriguingly, this method turned out to be the best gene selection method on the worst performing data set in this study, which also is the only data set without any significantly discriminatory genes (i.e. Sørlie, see Figure 4).

In this study, we selected methods that are commonly used to analyze gene expression data. Thus, although we see rather small difference between, for example, gene selection methods, this does not mean that gene selection is not important. Initially, we also included a gene selection method that rank genes based on variance, however, the consistently poor performance of this unsupervised method spurred us to omit it from further analyzes.

For comparing the performance of methods, we used the Wilcoxon signed rank test. This test considers the ranking of comparable pairs of method combinations (e.g. when comparing normalizations No 1 and No 2, combinations with equal choices for the other methods are paired, that is, the combination *No 1, G T-test, N 100, M SVM Linear *is compared to *No 2, G T-test, N 100, M SVM Linear*). Such paired tests are sensitive, and reveals interesting significant differences even when average performances are rather similar (i.e. Figures 3, 4, 5, 6). For individual methods, the Bonferroni correction was used to decide on statistical significance. However, when pairs of methods were considered, much fewer error rates were available to the statistical test, thus making methods for multiple hypothesis correction too insensitive. In these cases (Figures 8, 9, 10 and Additional files 3-4), we opt to use a fixed p-value threshold of 0.05 to identify interesting synergistic relationship, knowing that among these one must expect an increased number of false positives (se Methods for more details).

The data sets and class definitions investigated in this article have previously been used to compare unsupervised clustering methods [7]. There are some interesting parallels to be drawn between these two studies. First and foremost, both studies experienced huge differences in performance between data sets, and in particular the results from Sørlie did not agree with that of the other data sets. Normalization was shown to have a positive effect on both clustering and machine learning, but it was harder to draw any decisive conclusions about the relative performance of different normalization methods (i.e. No 1-4). Although the clustering study mainly focused on unsupervised gene selection methods, both studies found that relatively high numbers of genes were needed to obtain good performance.

## Conclusions

In this study, we have performed a comprehensive study of the effect that normalization, gene selection, the number of selected genes and machine learning method have on the predictive performance of resulting models. A unique aspect of this study was the inclusion of different normalization methods in the comparisons. Indeed, we showed that there is a significant positive effect of normalization on the best methods; however, the relative performance of different normalization methods is complex. The best machine learning methods in this study were Support Vector Machines (SVMs) with a radial basis kernel followed by SVMs with a Linear kernel and SVMs with a polynomial kernel of degree 2. We showed that there is a positive, synergistic relationship between these methods and gene selection based on the T-test and the selection of at least 150 genes.

## Methods

### Data

We used seven previously published 2-channel microarray experiments with a common reference design [23]. All data sets regard different types of human cancer. Five of the experiments used custom made arrays, while the last two used commercially produced arrays (Agilent). Since the design and normalization procedures of one-channel microarray experiments (e.g. Affymetrix) are not comparable with 2-channel experiments, they are not included in the study although they are widely used. The data sets contain from 40 to 133 samples each and are all published in high-impact journals.

Since the aim of this study was to investigate how the performance of various machine learning methods is affected by the choice of normalization method and gene selection, we must have true class partitionings to compare with the predicted classes from the models. To make it easier to compare the results for different models and data sets, we choose to divide data into two distinct classes:

**Alizadeh**: 133 samples corresponding to patients with different lymphomas, including patients with diffuse large B-cell lymphoma (DLBCL) [24]. Samples also include normal cell samples and a variety of cell lines. We define the two classes as DLBCL (68) and all other samples (65).

**Finak**: 66 samples profiled using Agilent microarrays [25]. We define the two classes according to the distinguishable tissue types; epithelial (34) and stromal tissue (32).

**Galland**: 40 samples profiled using Agilent microarrays [26]. The classes are defined as invasive (22) and non- invasive non-functioning pituitary adenomas (NFPAs) (18).

**Herschkowitz**: 106 samples. We define the two classes according to the level of ER expression; high (59) or low (47) [27].

**Jones**: 91 samples. We define the classes as the two most distinctive ones in the data set; the cancerous (72) and the non-cancerous (19) samples [28].

**Sørlie**: 73 samples. The classes are defined according to the level of ER expression; high (55) or low (18) [29].

**Ye**: 87 samples. We define the two classes as in the original study [30]; metastatic (65) and non-metastatic (22) patients.

Expression values in this study are the log_2_-ratios between the treated channel and the reference channel (M-values).

### Normalization

The normalization methods we investigated were the four possible combinations of two dye-normalization methods, global MA-loess [31] and print-tip (local) MA-loess [32], and two approaches to background correction, local correction [33] and no background correction. In addition, we also used non-normalized data (i.e. raw data):

**No 0**: Raw data (no normalization)

**No 1**: Print-tip (local) MA-loess, no background correction

**No 2**: Print-tip (local) MA-loess, background correction

**No 3**: Global MA-loess, no background correction

**No 4**: Global MA-loess, background correction

No 1 and 2 were not performed for the data sets Galland and Finak, since print-tip normalization is not relevant for the Agilent microarrays.

### Filtration and missing value imputation

In each of the seven data sets there were spots flagged by the scanner or the experimentalist, and some spots also had a lower signal than the background (in the cases where background correction was adopted). These were all marked as missing values. A discussion on how to treat flagged spots can be found in [34]. We chose to include samples with less than 50% missing values and genes with less than 30% missing values. Samples and genes were filtered simultaneously and the remaining missing values were imputed [35], [36]. There are several approaches to dealing with missing values; ROW imputation replace missing value with the row median (the median of that gene), while SVD (singular value decomposition) imputation uses a linear combination of the *k *most significant eigengenes to estimate missing values [37,38]. According to [7], these two approaches do not significantly affect the result of clustering analysis, and since we study the same data sets, we only use the SVD imputation in our study. We use the function svdImpute in the package pcaMethods to impute missing values.

In addition to the above described filtration and missing value imputation, we also remove some samples with incomplete annotations (i.e. class information). To avoid including duplicate genes we choose to compute the mean value of these.

### Cross validation and model evaluation

In this study we perform double cross validations; a 10-fold inner CV was used to optimize parameters in the methods and a 5-fold outer CV was used to estimate the final classification performance (see for example [38] for more on CV). The models were trained on the training data and evaluated on test data. The data partitioning was done so that the number of distinct patients in each test set differed by at most one patient. However, in data sets with several samples from the same patient (Ye, Alizadeh and Finak), all samples belonging to the same patient were always placed in the same test set, thus the number of samples per test set sometimes differed by more than one.

We used error rate, i.e. the percentage of misclassified observations in a test set, as a measure of classification performance. The error rates were adjusted by dividing by the theoretical error rate obtained by randomly assigning classes given the distribution of the two classes. Thus adjusted error rates below one correspond to models performing better than random class assignment. We performed the outer CV with 10 different random partitions into training and test sets. Thus the error rate of each combination of methods was estimated from 50 different test sets (10 5-fold CVs).

### Gene selection

In order to reduce the number of genes used by the machine learning methods, we always performed gene selection. There are several different types of gene selection techniques [8], but we decided to use three supervised approaches in this study:

**T-test**: A two sided, two sample t-test was used [37,39] and genes with the lowest p-values were chosen for further analysis. We used the function t.test from the package stats in R.

**Relief**: The Relief-algorithm assigns a value to each gene based on how well it separates nearest neighbors with different class labels [40]. We implemented a modified version of the function relief from the package dprep in R. The algorithm was modified so that all observations are chosen once rather than being sampled with replacement.

**Paired distance**: This is, to the best of our knowledge, a new gene selection approach based on investigating pairs of genes. We first chose the 1000 genes with the highest variance and the 1000 genes with the highest absolute mean value, and retained the union of these genes. For each of the chosen genes we then calculated two medians, one for those observations belonging to the first class and one for those observations belonging to the second class. In the two dimensional space spanned by the gene-pairs, we then calculated the Euclidian distance between the two corresponding pairs of medians. Finally, we ranked pairs of genes according to these distances.

The Decision trees (see Machine learning methods) also used an embedded technique for gene selection. Initially, we also used variance as an unsupervised gene selection method, but this strategy was later dropped due to its inferior performance.

Given the ranked lists from the gene selection methods, we selected 21 different numbers of genes to be used for inducing machine learning models (see Table 1). Some machine learning methods could not be run with all numbers of genes. In particular, SVMs could not handle only two genes in a satisfactory way, while NNs were not run with more than 150 genes for "one hidden layer" and 900 genes for "no hidden layer" due to computational costs.

### Machine learning methods

We use three different supervised machine learning methods; Support vector machines (SVMs), Artificial neural networks (NNs) and Decision trees (DTs). Most machine learning methods have several parameters that need to be set before inducing the model. To avoid overfitting the models, some of the most important parameters were optimized using an inner cross validation. Parameters not specifically mentioned were set to default values.

#### Support vector machines, SVMs

The idea behind SVMs is to use an implicit kernel function to map the data into an *n *dimensional space where the classes are separated by a hyperplane constructed to create the largest possible margin between the classes [38,41]. We use the function ksvm from the package kernlab in R to build SVM models (see [42] for a comparison of different SVM-packages in R). The data were not standardized, i.e. scaled to zero mean and unit variance (default in the ksvm function), before machine learning methods were applied. After some experiments with the different types of SVM classifications (C-svc, C-bsvc and nu-scv) we decided to use nu-scv for all the SVM models. We also experimented with different kernels and decided to use the following four kernels; linear, polynomial with degree 2, polynomial with degree 3 and radial basis kernel. The parameter nu was set to 0.2, which is the default value in ksvm. The inverse kernel width, sigma, for the radial basis kernel function was optimized using a grid search by choosing the value with the lowest cross validation error rate obtained from the ksvm function.

#### Artificial neural networks, NNs

NNs consists of connected units (neurons) that transform the input values to an output value based on a threshold function applied to the weighted sum of the input values [38,43,44]. We use two types of NNs; one with one hidden layer and one with no hidden layer (i.e. a perceptron). The number of units in the hidden layer was optimized in the inner cross validation (allowed values were 2, 3, 4 and 5). We use the function nnet from the package nnet in R to build NN models [45]. We set the maximum number of iterations to 100 and the value of rang to 0.1. After experimenting with different decay values we choose the value 0.001. Due to computational costs, models were only built from a maximum of 150 genes for one hidden layer and 900 genes for no hidden layer.

#### Decision trees, DTs

DTs are built by iteratively splitting the data using the most separating gene, thus forming a tree with nodes and leaves [38,43,44]. We used two different splitting criteria; information gain index and Gini index [46]. We used the function rpart from the package rpart in R to build the DT models. The trees were pruned by choosing the number of splits from the tree with the lowest cross-validated error [45].

Including settings (such as kernels in SVMs), we investigated the performance of the following eight machine learning methods:

**DT Gini**:                Decision tree with Gini index as splitting criteria

**DT Information**:   Decision tree with Information index as splitting criteria

**NN One layer**:      Artificial Neural Network with one hidden layer

**NN No layer**:        Artificial Neural Network with no hidden layer

**SVM Linear**:         Support Vector Machine with a linear kernel

**SVM Poly2**:          Support Vector Machine with a polynomial kernel of degree 2

**SVM Poly3**:          Support Vector Machine with a polynomial kernel of degree 3

**SVM Rb**:               Support Vector Machine with a radial basis kernel

### Analysis of results

The possible combinations of the different methods described above gave in total 14685 models with calculated error rates and corresponding standard deviations. To analyze these results, we used multiple linear regression models with error rate as response and Data set (S), Normalization (No), Gene selection method (G), Number of selected genes (N) and Machine learning method (M) (see Table 1) as predicting variables. We also included second order interaction terms in the regression models. When examining the data sets individually we removed Data set as predicting variable. We used the functions lm and anova (with default values of parameters) in the package stats in R to conduct the regression analyses. Test for significant predicting variables was done using a Bonferroni corrected threshold (0.05 divided by the number of variables: 10).

We used the Wilcoxon signed rank test to determine whether there was any significant difference between error rates obtained by different individual methods (Figure 7) and between pairs of methods (Figure 8, 9, 10). The tests were performed using the function wilcox.test in R. Significance was determined by a Bonferroni corrected threshold for individual methods and a threshold of 0.05 for pairs of methods and synergistic relationships. Entries (*i,j*) in the heat maps equal the the number of data sets where method (or pair of methods) *i *is significantly better than method (or pair of methods) *j*. The number of significant data sets for combinations including No 1 or No 2 was adjusted by multiplying by 7/5 since these methods were not applied to two data sets. The heat map was created with the function levelplot in the package lattice in R.

## Authors' contributions

JÖ was involved in developing the ideas presented in this article, implemented and conducted the machine learning, analyzed and interpreted the results and drafted the manuscript. EF collected the data sets, implemented filtration and missing value imputation and contributed in the general discussions. ML implemented and conducted the normalizations of the data. PR was involved in developing the ideas presented in this paper and interpreted the results. TRH was involved in developing the ideas presented in this article, analyzed and interpreted the results and wrote parts of the manuscript. All authors read and approved the final manuscript.

## Supplementary Material

Additional file 1 **Error rates obtained for all combinations of methods and data sets**. A table of the 14 686 different combinations of methods and data sets. Columns include Data set (D), Machine learning method (M), Gene selection (G), Number of selected genes (N), Normalization method (No), Error rate (E), Standard deviation of error rate (Esd), Adjusted error rate (Eadj) and Standard deviation of adjusted error rate (Esdadj). Click here for file

Additional file 2 **The predictive performance of individual methods across data sets**. Comparisons of the predictive performance of individual methods (data material for Figure 7). Click here for file

Additional file 3 **The predictive performance of pairs of methods across data sets**. Comparisons of the predictive performance of pairs of methods (data material for Figures 8-10 and additional comparisons). Click here for file

Additional file 4 **Synergistic relationships between pairs of methods across data sets**. The matrix show the number of data sets in which pairs of methods (given by one row and one column) performed significantly better (upper-right half, positive values) or significantly worse (lower-left half, negative values) than the best/worst method did individually. The Wilcoxon signed-rank test was used to compare the error rates of all combinations containing the pair against the error rates of all combinations containing the best/worst single method. Significance was determined using a p-value threshold of 0.05. Click here for file
